# Non-clinician involvement in interprofessional health sciences education: educator experiences and attitudes

**DOI:** 10.5195/jmla.2025.1763

**Published:** 2025-04-18

**Authors:** Rachel R. Helbing, Robert C. Hausmann

**Affiliations:** 1 rachel.helbing@cuanschutz.edu, Head of Health Sciences Libraries, Associate Librarian, University Libraries, University of Houston, Houston, TX; 2 rchausma@central.uh.edu, Director of Graduate Programs in Health Sciences, Clinical Full Professor, College of Education and Tilman J. Fertitta Family College of Medicine, University of Houston, Houston, TX

**Keywords:** Interprofessional education, facilitation, critical incident technique, qualitative research, inductive thematic analysis, collaborative practice

## Abstract

**Objective:**

The objective of this study was to assess educator views on the knowledge, skills, and abilities needed by IPE facilitators and to explore their attitudes toward and experiences with non-clinician facilitators of IPE activities, particularly health sciences librarians.

**Methods:**

This qualitative study utilized a novel questionnaire that included both multiple-choice and free-text questions. The latter were grounded in critical incident technique (CIT), a methodology that uses direct observations of human behavior to solve practical problems. The questionnaire was distributed electronically to the study's population of health sciences administrators, faculty, and staff in Texas who were involved with IPE. Multiple-choice data were analyzed via descriptive statistics, while free-text data were coded and analyzed via inductive thematic analysis principles.

**Results:**

There were 48 responses out of 131 individuals contacted directly for a response rate of 36.64%. Educators recognized a wide range of characteristics needed by IPE facilitators but viewed interpersonal skills as most important. While many reported experience with non-clinician facilitators of IPE activities, fewer had experience working with health sciences librarians in these roles. Educator attitudes toward non-clinician facilitators of IPE, including librarians, were largely positive.

**Conclusions:**

The findings of this study indicated that educators view interpersonal skills and the ability to elicit engagement as more important skills for IPE facilitators than a relevant clinical background. With proper facilitator training, non-clinicians could build upon their existing skillsets and increase their involvement with IPE, creating a larger pool of potential facilitators. A greater availability of skilled facilitators could increase the incidence of IPE, potentially resulting in more collaborative care and improved patient outcomes.

## INTRODUCTION

According to the World Health Organization (WHO), interprofessional education (IPE) “occurs when students from two or more professions learn about, from, and with each other to enable effective collaboration and improve health outcomes” with the goal that students who take part in IPE will be prepared for the kind of collaborative practice that can improve outcomes in real-world health care settings [1]. A recent review found that the top disciplines contributing to the IPE literature are medicine, nursing, pharmacy, dentistry, occupational therapy, and physical therapy [2]. While health sciences libraries have been deeply involved in health sciences education, particularly in evidence-based practice (EBP) 3–14] and the online learning arena 15–20], they have played a smaller role in the provision of IPE. This may be because most IPE activities are focused on clinical simulations and experiential training [21], where librarians' experience is less relevant.

**Figure 1:**
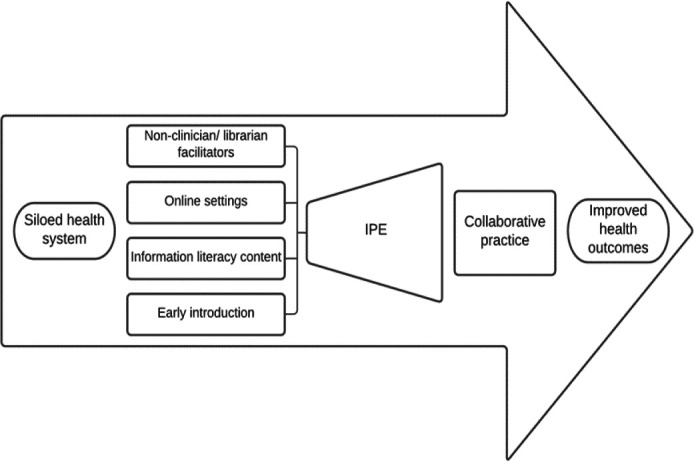
Strategies to Increase IPE Conceptual Framework

In 2010, WHO introduced a framework on the health and education systems that highlighted the importance of local context and IPE to build a collaborative practice-ready health workforce to strengthen the health system and improve health outcomes [1]. The WHO framework served as a starting point and inspiration for an updated conceptual framework informed by the results of this study. The new framework begins with the currently siloed health system. Utilizing non-clinician/librarian facilitators and online settings, incorporating lower-stakes learning content such as information literacy skills, and introducing interprofessional experiences early in the curriculum are all factors that could contribute to institutions increasing their offerings of IPE activities for students. In turn, these more robust IPE programs could potentially lead to stronger collaborative practice skills and ultimately improve health outcomes. Figure 1 displays this conceptual framework visually.

Focusing on the non-clinician/librarian facilitators facet of the framework, a search of the literature revealed limited documentation of direct librarian support for in-person IPE, including designing conferences, workshops, and continuing education modules 22–24]. Likewise, there are multiple reports of librarians planning and leading interprofessional book clubs 25–26]. At individual institutions, librarians have been involved in designing formal IPE programs for institutional staff and clinicians 27–29]. There are also a few examples of direct librarian involvement in online IPE activities that included online learning modules and tele-mentoring 30–33].

Librarians do not just support IPE activities; they also take part in interprofessional activities. While interprofessional teams are typically thought of as being made up of clinicians, there are examples in the literature of librarianship being considered one of the professions on an interprofessional team. This concept is not new. Clinical medical librarians have been involved in rounding since at least the 1970s, and they often round as members of interprofessional clinical teams, recognizing information needs and providing evidence to support clinical decision-making [34]. Another significant example of librarians serving as accepted members of interprofessional teams comes from the Interprofessional Education Collaborative's (IPEC) core competencies which list library science as one of the involved professions [35]. Further evidence of the legitimacy of this role for librarians has been provided in published reports of individual projects within academia [26, 30, 36].

In addition to the valuable contribution that librarians can make, libraries can also provide much needed space. Libraries have been described as interprofessional, collaborative spaces that bring different programs together [25, 37]. IPE, as it has traditionally been conducted, requires space. In order to facilitate and encourage IPE, faculty and administrators may need to look beyond their own departments' siloed spaces and find locations that are accessible and welcoming to all. Libraries can do this directly by holding IPE activities within their walls, or indirectly by purposely creating an environment conducive to serendipitous interprofessional interactions 37–38].

These examples that include librarian involvement make up only a small proportion of the overall literature on IPE and highlight the potential for librarians to contribute to health professions education in this area. The lack of literature on non-clinician IPE facilitators in general also underscores the need to develop an understanding of educator views on IPE facilitation. Identifying the perceived facilitator characteristics that lead to successful IPE activities can illuminate a path forward to increased librarian involvement. In turn, deepening the pool of potential facilitators can increase the number of meaningful IPE experiences available to students during their health professions education. This study was designed to assess educator views on the knowledge, skills, and abilities needed by IPE facilitators and to explore their attitudes toward and experiences with non-clinician facilitators of IPE activities, particularly health sciences librarians.

The following research questions guided this study:

1. What knowledge, skills, and abilities do health sciences educators deem necessary for facilitators of IPE activities
2. What are health sciences educators' experiences with and attitudes toward non-clinician facilitators of IPE activities
3. What are health sciences educators' experiences with and attitudes toward health sciences librarians in particular as facilitators of IPE activities

## METHODS

### Instrument

This study used a novel questionnaire designed to collect data which addressed the posed research questions. No existing validated tools addressed this study's specific research questions, necessitating the creation of a new questionnaire. The questionnaire consisted of a mix of multiple-choice and free-text entry questions. Multiple-choice questions captured demographic data and length of experience with IPE. Additional questions asked about experience with non-clinician facilitators of IPE. Attitude-measuring questions used five-point Likert scales and were designed to measure participants' attitudes toward non-clinician IPE facilitators. Specifically, two of the questions asked participants if they thought non-clinicians, and librarians in particular, possess the ability to facilitate in-person IPE. The answer choices ranged from “Not at all” to “A great deal.” The other two questions asked if participants were willing to collaborate with non-clinicians, and librarians in particular, on IPE facilitation in the future. The answer choices ranged from “Unwilling” to “Willing.”

The remaining data were collected via free-text entry questions. Some of these questions were adapted from critical incident technique (CIT), a methodology that uses direct observations of human behavior to solve practical problems. A key feature of CIT is asking research participants to recall and describe a time when the phenomenon of interest occurred. This occurrence of the phenomenon of interest is the “incident.” Framing the question in this way is intended to improve recall and provide more specific and relevant data [39]. CIT can be used as a methodology to determine what factors help or hinder a particular activity [40]. CIT was used in this study to examine participants' experiences with non-clinician facilitators of IPE. The participants were asked to recall a time when they participated in an IPE activity that included non-clinician facilitators and then share more about that experience.

The remaining free-text entry questions directly asked research participants about characteristics needed by IPE facilitators and the rationales behind their levels of willingness to collaborate with non-clinician IPE facilitators (and librarians in particular) in the future. The questions were ordered such that these questions appeared subsequent to the questions that asked participants to recall a time when they participated in IPE with non-clinician facilitators. This was to prime the participants to base their responses on any past relevant incidents they have experienced, taking further advantage of the CIT method.

The reliability of this study was strengthened through Robson and McCartan's principles of avoiding common pitfalls in data collection such as transcription errors and using an audit trail to show others that the research has been carried out thoughtfully, carefully, and honestly [41]. Validity was strengthened through triangulation, peer debriefing and support, negative case analysis, and the use of an audit trail [41].

### Participants

The population for this study consisted of health sciences administrators, faculty, and staff in the state of Texas who were involved with IPE. The study participant sample was primarily drawn from the subpopulation of members of the Texas IPE Consortium (TX IPE), a group formed in 2015 by leadership in academic health sciences centers located in the state of Texas to “foster cross-institutional collaboration in order to expand learning opportunities and reinforce value for IPE as a critical aspect of health professions education” [42].

The individual members of the TX IPE were contacted via email with a link to the online questionnaire. The email was also shared with the TX IPE listserv and forwarded to faculty and staff involved in Texas Educators Academies Collaborative for Health Professions-Southeast (TEACH-S). Additionally, a link to the questionnaire was shared in the chat of a virtual IPE summit that was held during the data collection period and had been promoted throughout Texas. The participants in this study constituted a purposive sample, as the TX IPE members were targeted in a nonrandom manner to represent a cross-section of the larger population of educators involved with IPE in Texas.

### Procedures

The University of Houston Institutional Review Board reviewed this study and determined it was exempt on October 14, 2021.

The questionnaire was constructed and distributed via Qualtrics, a web-based survey platform. The questionnaire was opened and disseminated successfully via email to 116 out of 131 individual members of TX IPE with valid email addresses on October 18, 2021. The questionnaire link was also provided to the TX IPE listserv, faculty and staff members of TEACH-S, and the attendees of a virtual IPE summit in October and November 2021. The supplementary groups largely consisted of the same individuals as the TX IPE membership. Reminder emails were sent to TX IPE members once. The questionnaire remained open for 30 days. Responses were anonymous, and no compensation was provided for participation in the study.

The majority of the data analysis in this study focused on the categorical data obtained from the CIT-based free-text entry questions. This was conducted via inductive thematic analysis principles [43]. As this study was examining an emerging area, it was not appropriate to identify themes prior to data collection and analysis; inductive thematic analysis ensured the themes were grounded in and emerged from the data. The researcher coded the responses line-by-line and pooled into themes the critical incidents; knowledge, skills, and abilities; and rationales identified. These themes were further organized under broader domains to create frameworks which were explored narratively.

## RESULTS

There were 48 responses, resulting in a response rate of 36.64% of the 131 individual TX IPE members. The responses to the questionnaire's demographic items showed an experienced and diverse set of study participants. Most (61.29%) reported a faculty status of assistant, associate, or full professor, with assistant professor being the most frequent response (25.81%). Over two-thirds (70.97%) of the participants reported being a practicing clinician, either currently or in the past. Among the clinicians, the most common clinical fields reported were Nursing (22.73%), Counseling (18.18%), and Physical Therapy (13.64%), with those three combined making up over half the responses (54.55%). Appendix B provides a graphical representation of the participants' fields of clinical practice.

Most participants (63.04%) reported being very experienced in IPE, indicating six or more years of involvement. Very few participants (6.52%) indicated less than one year or no experience with IPE. Additionally, more than half (55.88%) indicated that they had taken part in IPE with non-clinician facilitators. Nearly half (46.15%) of these non-clinician facilitators were administrative staff. Only two (7.69%) participants reported experience with librarian facilitators. Additionally, two (7.69%) participants reported experience with students taking roles in IPE facilitation.

### Knowledge, Skills, and Abilities Required for IPE Facilitation

All participants, regardless of whether they had experience with IPE that included non-clinician facilitators, were asked to provide free-text feedback on the knowledge, skills, and abilities required for in-person IPE facilitation. There were responses to this question from 30 participants. These responses were analyzed in order to develop a framework on IPE facilitators. The responses to this question revealed that interpersonal skills were valued above other areas including knowledge and management skills.

The ***Interpersonal Skills***domain ranked highest with the ability to elicit discussion and participation from all students being the most frequently cited necessary skill. Participants mentioned the need to “draw in students who are not participating in discussion,” and to encourage and guide participation. The importance of guiding the conversation without monopolizing it and listening rather than teaching were also emphasized. Additionally, several participants specifically mentioned creating an environment of “psychological safety.” One participant summed up the importance of this domain in writing, “So much of IPE is about communication and teamwork, not clinical knowledge.”

For the ***Knowledge***domain, participants cited the need for the planners and facilitators of IPE to represent a variety of professions, and thus have personal knowledge of interprofessional work while also modeling it. While some participants wrote that facilitators must have “expert knowledge” of the content being covered, others specified that only a “basic knowledge of the topic at hand” was needed and that the facilitator “does not have to be an expert in the content.” Many of the responses focused on knowledge of the planned IPE activity or knowledge of the participating health professions' roles and responsibilities, things that could be taught to facilitators of any background during a training session. Other participants specifically called out knowledge that must be obtained through clinical experience.

**Figure 2:**
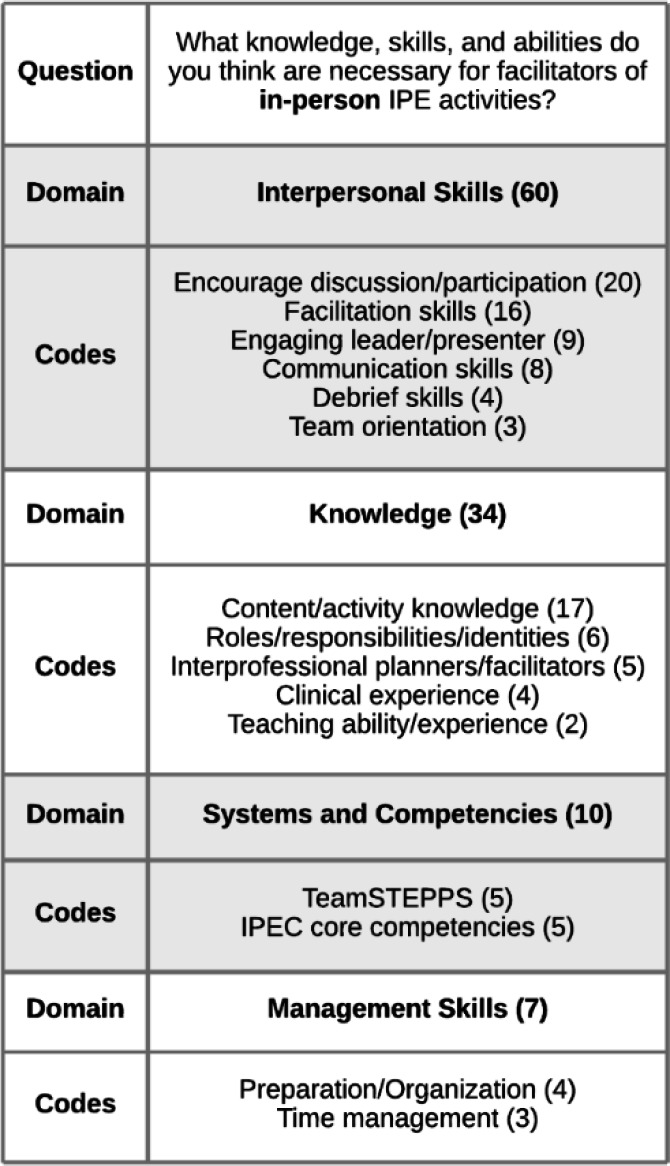
IPE Facilitators' Needed Knowledge, Skills, and Abilities Coding Framework

***Systems and Competencies***was made up of the pre-packaged TeamSTEPPS® curriculum and the IPEC core competencies document, which were referenced as tools that should be utilized by IPE facilitators from all backgrounds. Participants also infrequently mentioned ***Management Skills***, including preparation, organization, and time management. Figure 2 above provides a complete listing of this framework's domains, individual codes, and their frequencies.

### Success Factors for IPE with Non-Clinician Facilitators

Of the 34 participants who reported having participated in an IPE activity that included non-clinician facilitators, 13 (38.2%) provided written responses to the free-text questions that asked them to describe the factors contributing to the IPE activities' success or lack thereof. These written responses were analyzed in order to construct a framework on the success factors for IPE with non-clinician facilitators.

***Designing for Engagement***emerged as the top domain, with participants highlighting the need for “dynamic/compelling activities for students” that should also be clinically relevant, small groups to encourage discussion, and a good interprofessional mix of students. One participant pointed out that having students lead the IPE activity naturally led to high levels of student engagement. Next, it was shared that having enthusiastic, well-trained ***Strong Facilitators***from a variety of professions led to success.

Additionally, having ***Engaged Students***who actively participate, ***Effective Planning***(well-designed curriculum, utilizing support people throughout) and ***Successful Technology***(utilizing technology tools effectively, being familiar with the online platform, and having technology function during the activity) were mentioned as success factors.

The data that were coded on the nonsuccess side of non-clinician facilitator IPE resulted in four domains. ***Problems With Facilitators*** was mentioned most often of any nonsuccess factor. One participant stated that “non-clinicians struggle to connect with the clinical students. Their energy level and learning points don't always ring true for what is happening in the simulation…or in real life.” It was also mentioned that facilitators could be unprepared or lack skills or buy-in. One response discussed the difficulty with training facilitators from areas that had high turnover at the institution.

The next domain detracting from the potential success of IPE with non-clinician facilitators was ***Lack of Student Engagement***. It was brought up that students may have been unwilling to participate or lacked the knowledge and experience to participate meaningfully. Notably, a participant wrote that “some students did not respect staff being facilitators and they did not fully participate.” Completing the nonsuccess framework were ***Ineffective Planning***(scheduling problems and too many participants) and ***Technical Issues***.

**Figure 3:**
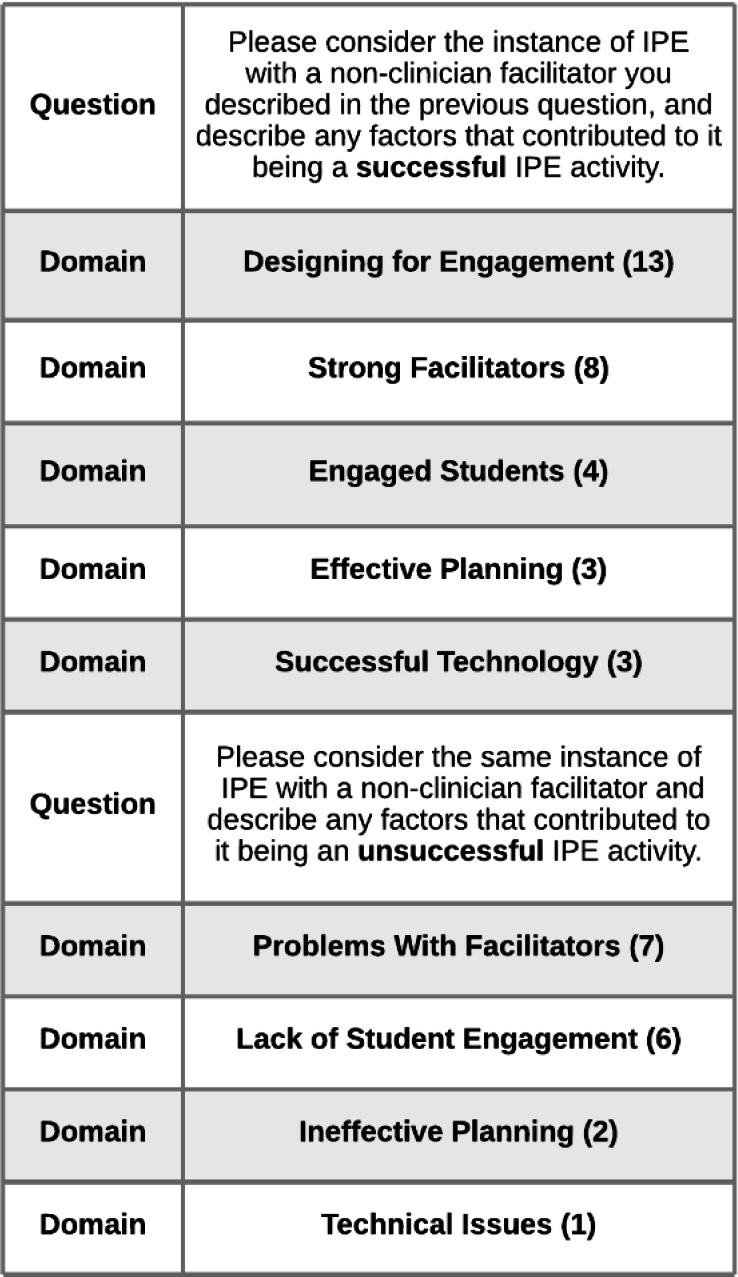
IPE with Non-Clinician Facilitators Success Factors Coding Framework

This framework demonstrated the importance of engagement in successful IPE activities that include non-clinician facilitators, as well as the need for facilitator training to produce strong facilitators who will not detract from the event's value. It also indicated that non-clinician facilitators may not be appropriate in all roles and/or all types of IPE activities. Figure 3 provides a complete listing of the code domains and frequencies that emerged from the data on success factors for IPE with non-clinician facilitators.

### Attitudes Toward Non-Clinician IPE Facilitators

All participants, regardless of previous experience, were asked questions to elicit their attitudes toward non-clinicians generally, and librarians in particular, as facilitators of in-person IPE activities.

**Figure 4:**
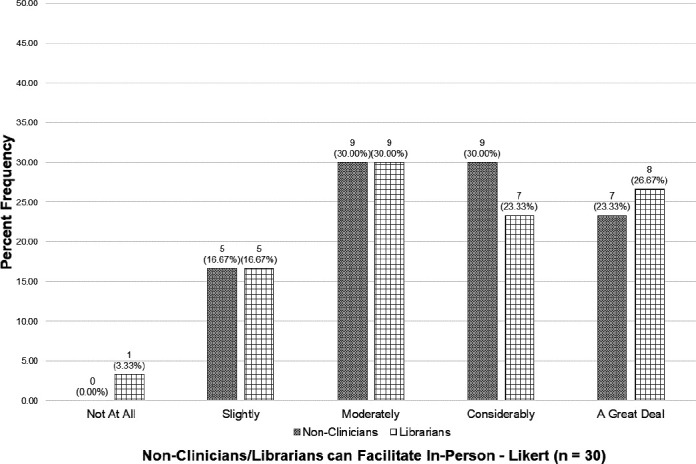
Attitudes Toward Non-Clinician and Librarian In-Person IPE Facilitation

When asked to rate to what degree they felt non-clinicians and librarians possessed the characteristics necessary to successfully facilitate in-person IPE, the large majority (83.33% for non-clinicians; 80.00% for librarians) chose at least *moderately*, with *moderately* being the most frequently chosen response. No (0.00%) participants chose *not at all* for non-clinicians and only one (3.33%) chose *not at all* for librarians. Figure 4 displays the complete responses to this question.

When asked to rate their willingness to collaborate with non-clinicians to facilitate IPE, the vast majority (93.55%) chose at least *somewhat willing*, with *willing* being the most frequently chosen response. No (0.00%) participants chose *unwilling* and only two (6.45%) chose *somewhat unwilling*.

**Figure 5:**
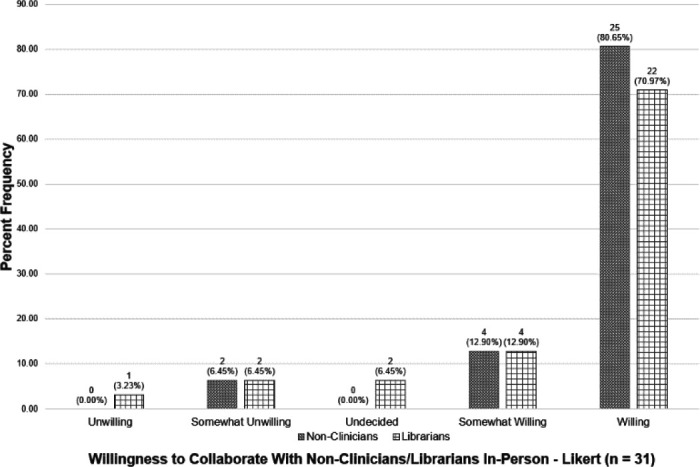
Willingness to Collaborate with Non-Clinicians/Librarians on In-Person IPE

When asked to rate their willingness to collaborate with librarians in particular to facilitate IPE, the responses were slightly less positive. Still, the large majority (83.87%) chose at least *somewhat willing*, with *willing* being the most frequently chosen response. There were three (9.68%) participants who chose *unwilling* or *somewhat unwilling*. Figure 5 displays the complete responses to this question.

The study's two final free-text questions provided participants with the opportunity to share the rationales for their levels of willingness to collaborate with non-clinicians and librarians on IPE facilitation. There were 27 participants who responded to these questions. Coding this data resulted in a framework on willingness to collaborate with non-clinicians/librarians on IPE that was largely focused on knowledge and skills as well as professional roles.

The top domain by frequency of coding for willingness to collaborate with non-clinicians was ***Knowledge/Skills***. Participants called out the potential for non-clinicians to possess valuable expertise and be skilled in communication, database searching, EBP, technology use, and more. Opinions on whether clinical experience was a help or hindrance were mixed, with some participants stating that “no history of clinical experience is not acceptable” and that they “feel they need to understand clinical practice to be totally effective,” while others wrote that “the purpose of facilitating is not to know the answers but to guide the activities/discussion” and “knowledge and skills related to teaching/engagement are more important than clinical experience.” One participant went so far as to write, “I think that it would be an advantage if the facilitator did not have any knowledge or skill in the fields of the participants." Another participant pointed out that many gaps in non-clinicians' knowledge could be filled with training.

The next most oft-cited domain in this framework was ***Professional Roles***. Some participants noted that non-clinicians could contribute to IPE in ways that reflected their support roles in clinical practice. Other comments from participants pointed out that they themselves were non-clinicians who facilitate IPE, and as such felt confident that other non-clinicians could carry out the same work. Some participants noted that students may lack buy-in when working with non-clinician facilitators, and it may be necessary for clinical students to work with clinician facilitators while non-clinical students work with non-clinician facilitators. Several responses noted the necessity for clarity of roles and self-awareness.

In the ***Collaboration***domain, participants wrote about the fact that non-clinicians are part of the interprofessional team and thus should be included in IPE. One mentioned advocating for “big tent inclusion” of non-clinician professionals in IPE, while another stated that it simulates the “real world” of frequent collaborations with non-clinicians. Additionally, librarianship was lauded as a particularly collaborative field.

Finally, the least-cited domain was ***Need for Interprofessional Mix***. One participant focused on the idea that “the more diversity of skills, ideas, backgrounds, the better!” with another stating “We need all the help we can get!!!” It was stated that librarians “bring a broader perspective across different health care entities” and “a different perspective that clinicians do not have.”

**Figure 6:**
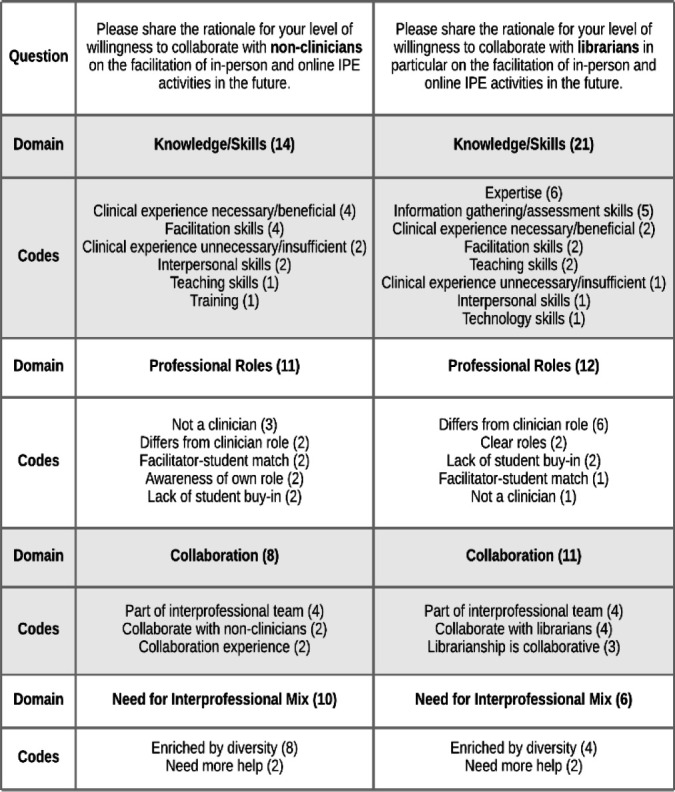
Willingness to Collaborate with Non-Clinicians/Librarians on IPE Coding Framework

This framework demonstrated the value of the diversity of knowledge and skills held by individuals from different professions. The responses largely showed support for non-clinician and librarian future involvement in IPE, although they included some mixed opinions on the necessity for clinical experience, again making the case that non-clinician facilitators may not be appropriate in all roles and/or all types of IPE activities. Figure 6 displays a complete listing of this framework's domains, individual codes, and their frequencies.

## DISCUSSION

The results of this study show that interpersonal skills and knowledge are highly valued in IPE facilitation. In particular, when asked what characteristics are necessary for IPE facilitators, the top responses were focused on encouraging discussion and participation, facilitation skills, and content/activity knowledge (see Figure 2). Additionally, Figure 3 shows that designing engaging IPE activities that are skillfully facilitated can lead to their success. Librarians who are already finding success in supporting IPE at their institutions through designing professional development programming [22–24, 27–29], interprofessional book clubs 25–26], and online learning modules 30–32] are likely skilled facilitators who elicit discussion and participation from participants while being knowledgeable about the professions and content involved. Going forward, librarians seeking to make inroads with IPE at their institutions can concentrate on building and showcasing their proficiency in these areas.

A small subset of participants' responses in this study underscored a lack of familiarity with the profession of librarianship. Librarians frequently encounter this lack of understanding from both their colleagues and the public, necessitating a continuous and proactive effort on their part to communicate the important leadership role librarians can have in IPE. These responses serve as a powerful reminder of the imperative for librarians to engage in active advocacy, effectively articulating their professional competencies and the significant contributions they make at their institutions. By actively promoting the profession and highlighting their diverse skill set, librarians can bridge the perceptual divide and ensure that their leadership role is recognized.

The purpose of this research was to explore one arena in which the collaborative and inclusive nature of librarianship can be leveraged for the benefit of health professions education, and the results were encouraging. While not true of all IPE educators, most view non-clinicians and librarians as skilled colleagues who possess many of the characteristics needed to effectively facilitate IPE (see Figures 4 and 6) and have a willingness to collaborate with them on this work (see Figure 5). The health professions educational landscape is primed for librarians to take on a leadership role in IPE through coordination, collaboration, and facilitation.

With these results in mind, health sciences programs should consider utilizing non-clinician IPE facilitators as brokers of knowledge between discipline-based experts. In utilizing non-clinicians, they should recruit those with strong interpersonal skills over professional discipline-based experience and knowledge. Potential facilitators include non-clinical faculty, administrative staff, instructional designers, librarians, and upper-level students. Training can ensure they are familiar with the roles of the professions involved and the planned activity in order to help secure success. Resources geared toward non-traditional facilitators and learning modalities can be utilized to build facilitator training programs that emphasize interpersonal skills and the effective use of technology 44–45].

Programs should identify more mechanisms through which non-clinicians can support, empower, challenge assumptions, and enable discipline-based professionals in discovering new approaches. In addition to supporting IPE by creating web-based information guides and providing journals and books on IPE, non-clinicians could search the literature to find cases to be used in the activity.

Additionally, consider incorporating activities that are less clinically focused, such as information literacy/EBP workshops, into the institution's IPE portfolio. This would help to ensure that students from different programs have similar baseline levels of skills [31] while enabling them to interact interprofessionally. Another option is interprofessional book clubs 25–26]. These possibilities would allow IPE to be introduced early in the curriculum while enabling a wider range of individuals to participate as facilitators.

Moreover, non-clinicians working in health sciences educational programs should feel empowered to approach the team in charge of IPE at their institutions and offer to help lead the change. Librarians should make the case that interpersonal skills and engagement are as important as clinical skills. Participating in the provision of IPE can also benefit the non-clinicians or librarians in terms of the opportunities for outreach and connections, widening and strengthening the understanding of their leadership role.

Many IPE activities continue to take the form of simulations which are heavily focused on high-stakes clinical content. A partial realignment of this focus could enable institutions to provide more robust IPE programs in order to better prepare their students for real-world collaborative practice. This study's results highlighted the importance of interpersonal skills and communication for IPE. Additionally, they made it clear that engagement is the most important factor contributing to IPE's success. IPE does not need to be limited to simulations of high-stakes clinical scenarios. Engaging activities can help students build interpersonal skills outside of the clinical simulation or case-based IPE paradigm.

The future of IPE should include more programs that are based on incorporating non-clinician and librarian facilitators, utilizing online settings for learning activities, teaching information literacy content, and introducing IPE experiences early in the curriculum. Increasing these factors would enable institutions to provide more robust IPE programs, allowing students to build solid foundations of interpersonal skills for collaborative practice, working up to the clinical simulations necessary for clinical learning later in the curriculum. If designed thoughtfully, conducting learning activities on library skills with interprofessional student teams would provide opportunities for students to build interprofessional communication skills in engaging formats. This strategy would introduce efficiencies while overcoming the barriers to large-scale clinical simulation-based IPE, allowing institutions to increase the number of IPE activities offered.

## LIMITATIONS

Since all study participants were likely Texas residents, the study was not representative of other geographic regions. As there was a low response rate to demographic questions, and participants were not asked about race, ethnicity, gender, or socioeconomic status, the diversity of the pool of participants in these areas could not be examined for representativeness of the population; thus, the results were not generalizable. Additionally, there were not enough responses to the demographic questions to make meaningful statistical comparisons between demographic groups. There were few participants who reported experience working with librarian facilitators of IPE, somewhat limiting the direct applicability of this study to librarians. Since responses were anonymous, the researchers could not follow up with participants to seek clarification or more information. This study was conducted as part of an author's doctoral research with limited resources and only one coder. The lack of a second coder for the free-text question responses detracted from the study's reliability. Finally, the focus on attitudes and experiences rather than outcomes assessment means this study serves as a starting point to inform future research in that area.

## Data Availability

Data associated with this article are available in the University of Houston Dataverse Repository at https://doi.org/10.18738/T8/IDIZOM.
