# Supplementary figures and images for: Non-clinician involvement in interprofessional health sciences education: educator experiences and attitudes
Source: J Med Libr Assoc. 2025 Apr 18;113(2):133–42. doi: 10.5195/jmla.2025.1763 (PMC12058338; doi:10.5195/jmla.2025.1763)

Appendix A. Complete Questionnaire.


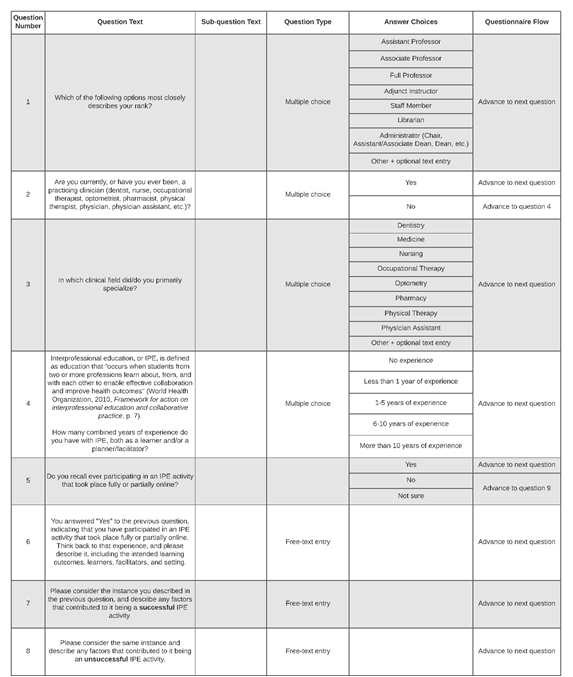


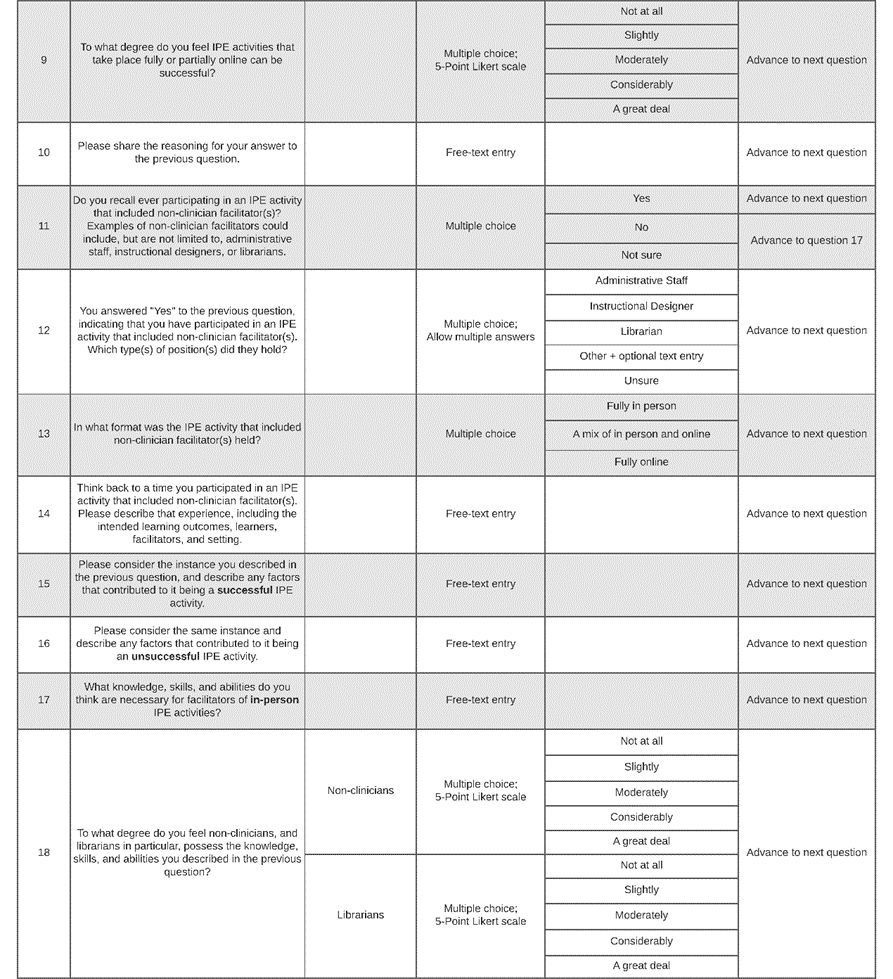


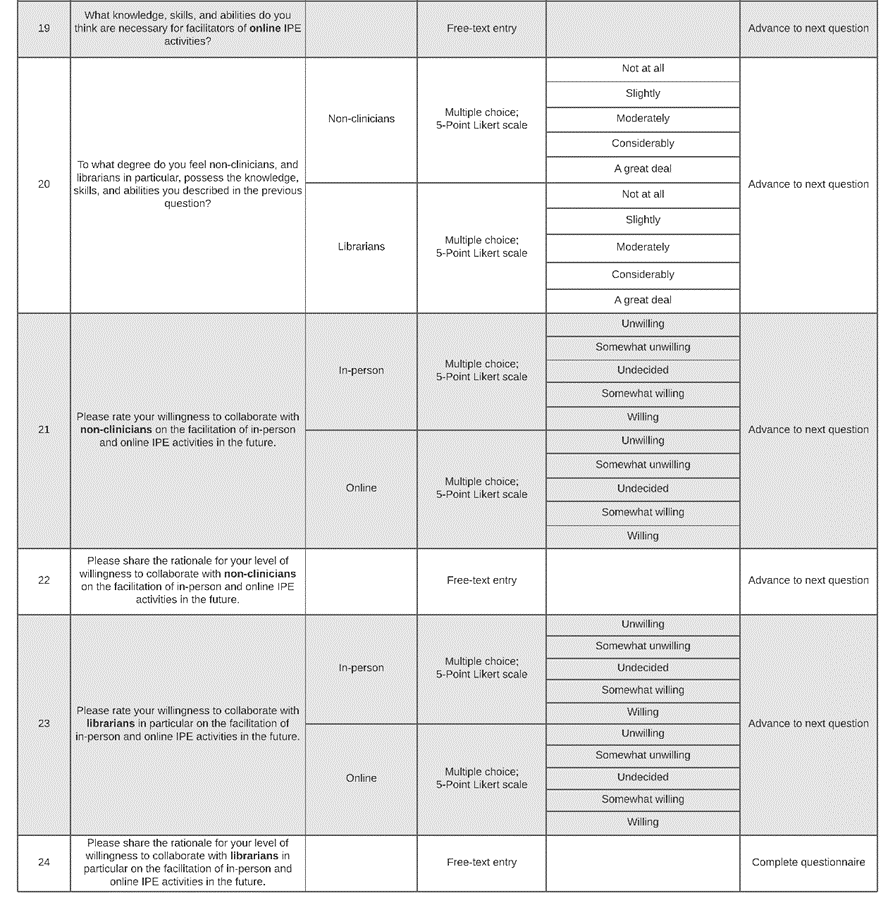

Supplement: Supplementary file 1 — Appendix A [file jmla-113-2-133-s01.docx]

Appendix B. Participants’ Fields of Clinical Practice.


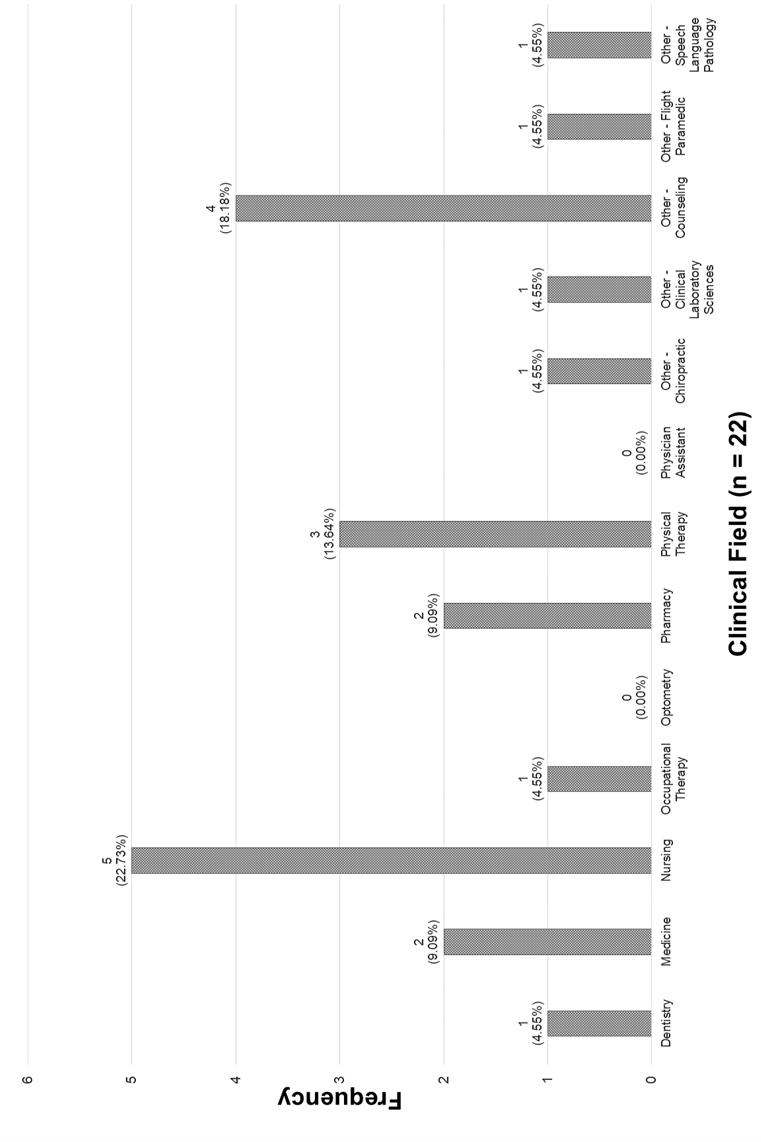

Supplement: Supplementary file 2 — Appendix B [file jmla-113-2-133-s02.docx]
